# Paleoseismic study of the Kamishiro Fault on the northern segment of the Itoigawa–Shizuoka Tectonic Line, Japan

**DOI:** 10.1007/s10950-016-9629-x

**Published:** 2016-11-21

**Authors:** Aiming Lin, Mikako Sano, Maomao Wang, Bing Yan, Di Bian, Ryoji Fueta, Takashi Hosoya

**Affiliations:** 1 Department of Geophysics, Graduate School of Science, Kyoto University, Kyoto, 606-8502 Japan; 2 School of Earth Sciences and Engineering, Nanjing University, Nanjing, 210046 China; 3 Chuo Kaihatsu Corporation, Saitama, 332-0035 Japan

**Keywords:** 2014 M_w_ 6.2 Nagano earthquake, Palaeoseismicity, Kamishiro fault, Recurrence interval, Morphogenic earthquake, Plate boundary

## Abstract

The M_w_ 6.2 (Mj 6.8) Nagano (Japan) earthquake of 22 November 2014 produced a 9.3-km long surface rupture zone with a thrust-dominated displacement of up to 1.5 m, which duplicated the pre-existing Kamishiro Fault along the Itoigawa–Shizuoka Tectonic Line (ISTL), the plate-boundary between the Eurasian and North American plates, northern Nagano Prefecture, central Japan. To characterize the activity of the seismogenic fault zone, we conducted a paleoseismic study of the Kamishiro Fault. Field investigations and trench excavations revealed that seven morphogenic paleohistorical earthquakes (E2–E8) prior to the 2014 M_w_ 6.2 Nagano earthquake (E1) have occurred on the Kamishiro Fault during the last ca. 6000 years. Three of these events (E2–E4) are well constrained and correspond to historical earthquakes occurring in the last ca. 1200 years. This suggests an average recurrence interval of ca. 300–400 years on the seismogenic fault of the 2014 Kamishiro earthquake in the past 1200 years. The most recent event prior to the 2014 earthquakes (E1) is E2 and the penultimate and antepenultimate faulting events are E3 and E4, respectively. The penultimate faulting event (E3) occurred during the period of AD 1800–1400 and is associated with the 1791 M_w_ 6.8 earthquake. The antepenultimate faulting event (E4) is inferred to have occurred during the period of ca. AD 1000–700, likely corresponding to the AD 841 M_w_ 6.5 earthquake. The oldest faulting event (E8) in the study area is thought to have occurred during the period of ca. 5600–6000 years. The throw rate during the early Holocene is estimated to be 1.2–3.3 mm/a (average, 2.2 mm/a) with an average amount of characteristic offset of 0.7–1.1 m produced by individual event. When compared with active intraplate faults on Honshu Island, Japan, these slip rates and recurrence interval estimated for morphogenic earthquakes on the Kamishiro Fault along the ISTL appear high and short, respectively. This indicates that present activity on this fault is closely related to seismic faulting along the plate boundary between the Eurasian and North American plates.

## Introduction

**Fig. 1:**
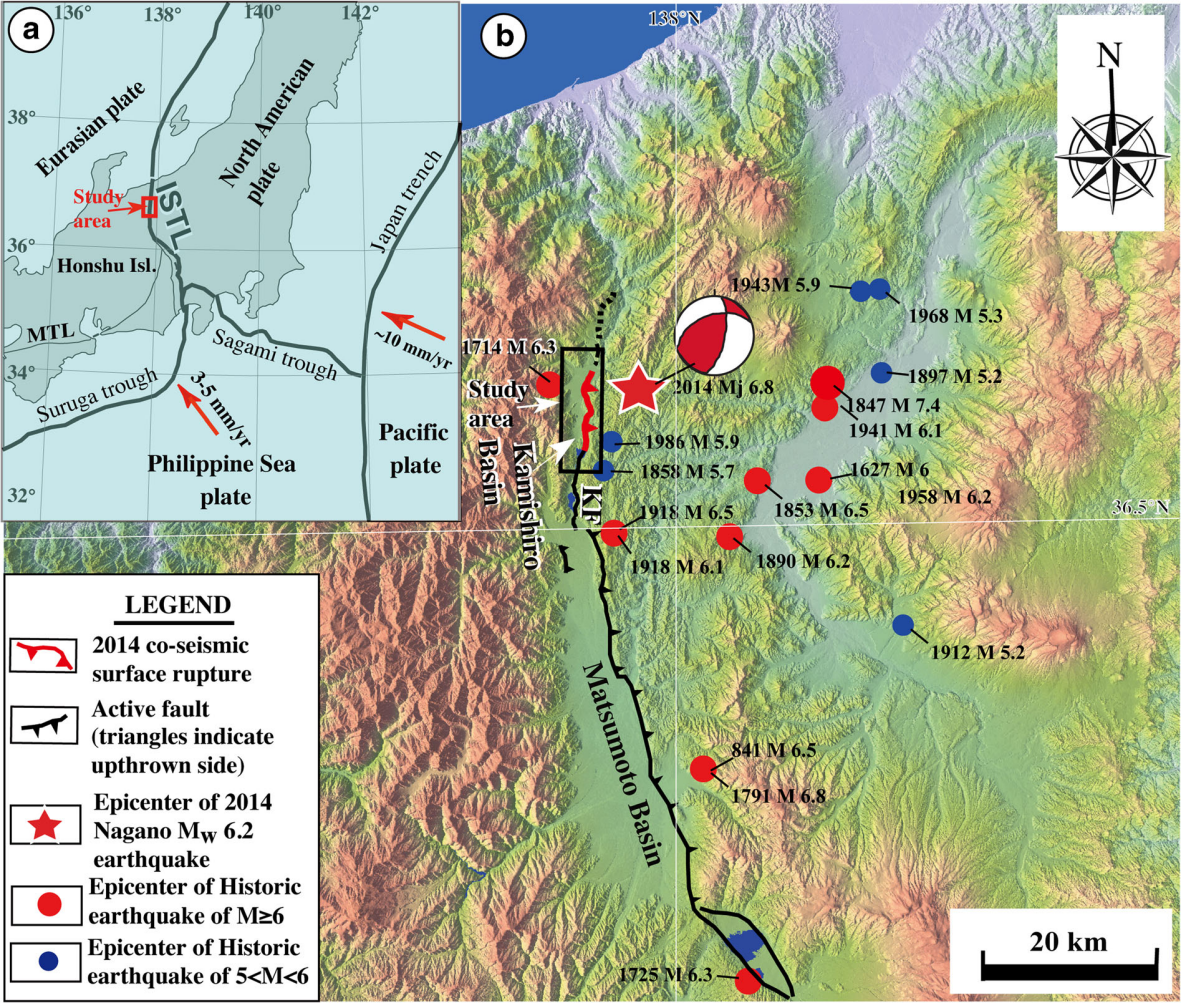
**a** Index map showing the tectonic setting of the study area. *MTL* median tectonic line, *ISTL* Itoigawa–Shizuoka Tectonic Line. **b** Color-shaded relief map showing the distribution of the Kamishiro Fault and M_w_ >5 seismicity in the study area around the Matsumoto Basin. Active fault data are from RGAFJ (1991). Epicenter data are from Geospatial Information Authority of Japan (2014) and Headquarters for Earthquake Research Promotion (2000). The focal mechanism of the 2014 M_w_ 6.2 earthquake is from Yamanaka (2014)

**Fig. 2:**
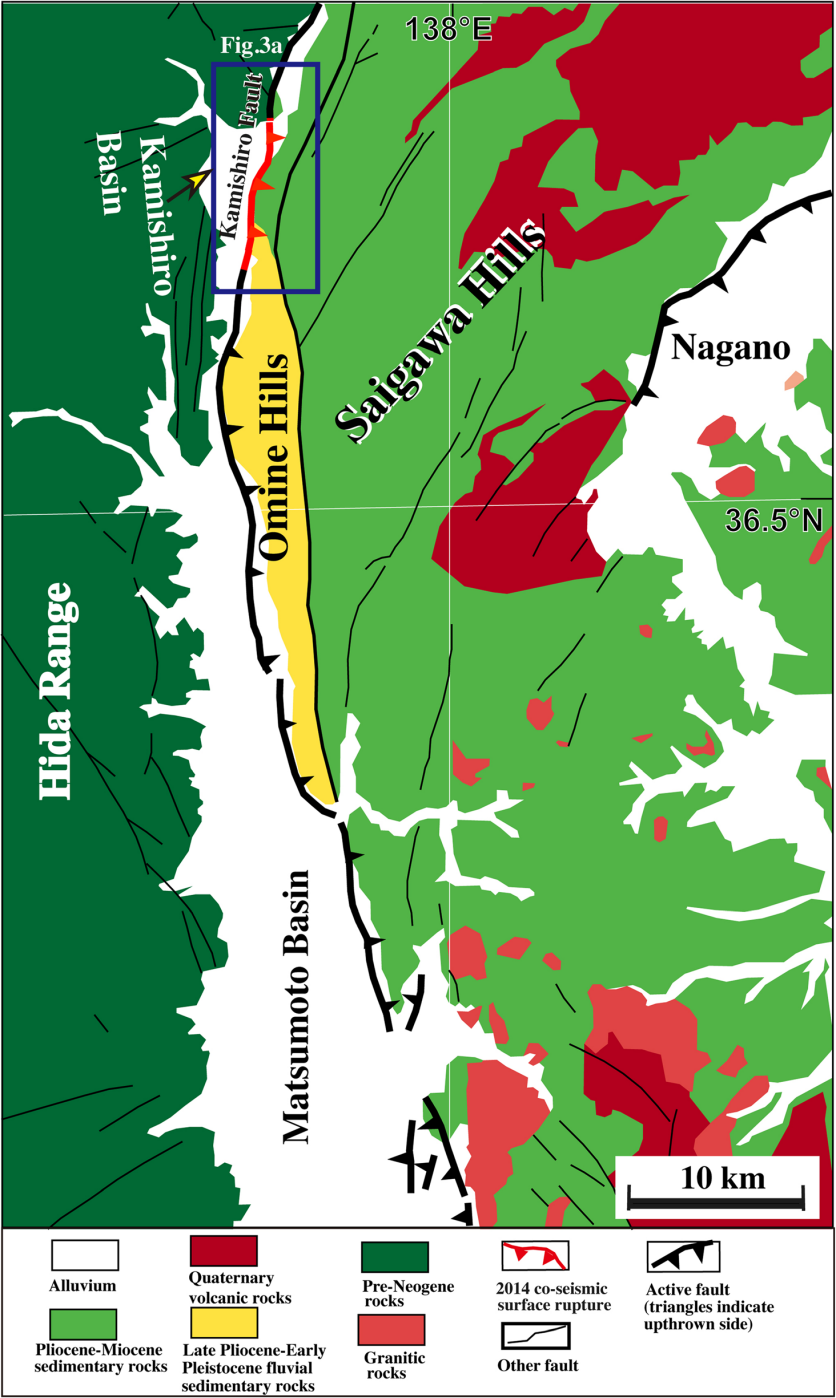
Geologic map of the study area around the Matsumoto Basin area. Modified from Nakano et al. (2002)

**Fig. 3:**
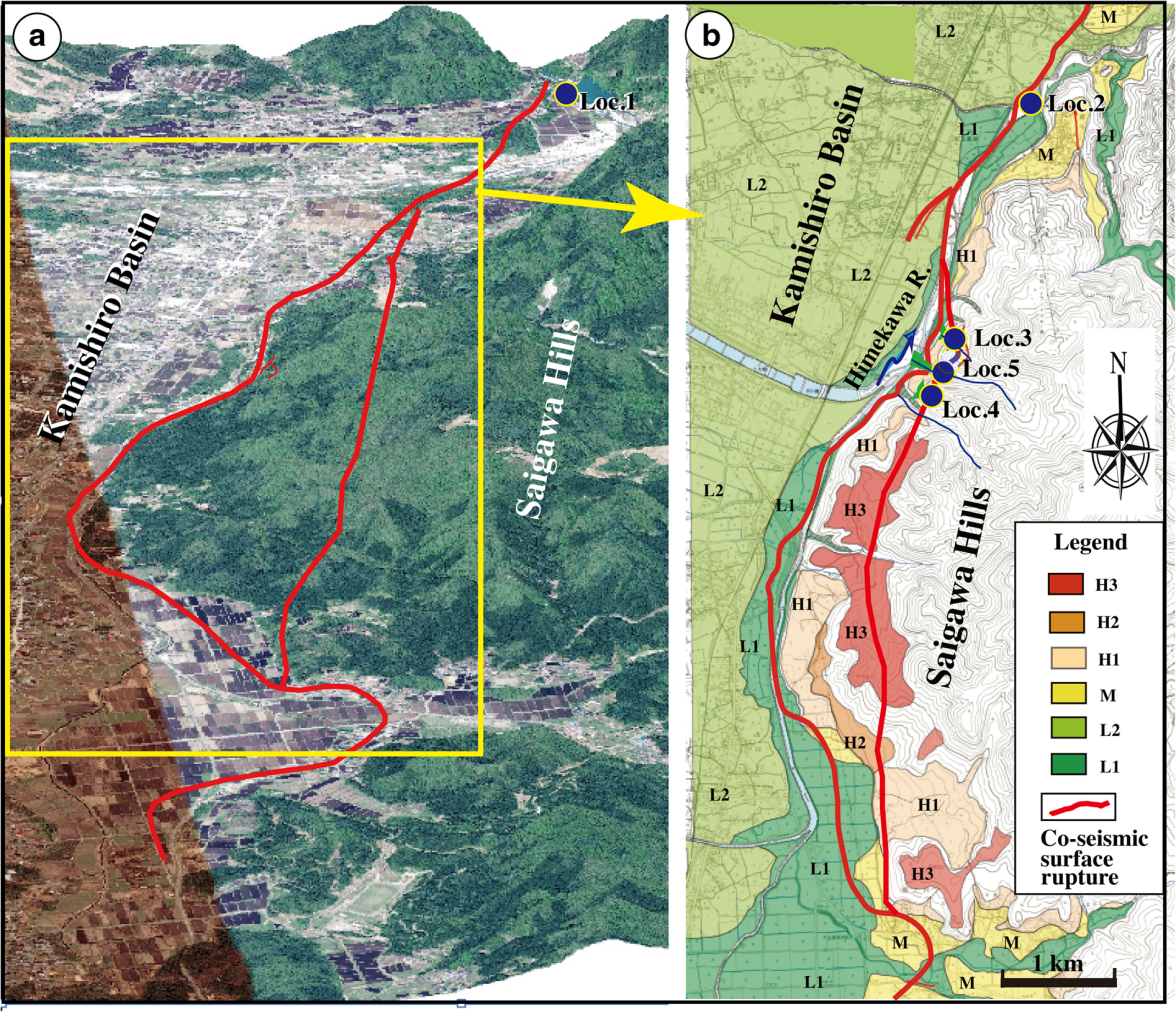
Topographic maps showing the topographic features and distribution of the 2014 co-seismic surface ruptures. **a** Color-shaded relief map showing a perspective view of topographic features in the study area and the distribution of the 2014 Nagano co-seismic surface ruptures. **b** Topographic map showing the distribution of terrace risers. Active fault data are from RGAFJ (1991). *H3* high terrace 3 (highest terrace), *H2* high terrace 2, *H1* high terrace 3, *M* middle terrace, *L2* lower terrace 2, *L1* lower terrace1 (lowest terrace). For details of the location, see Fig. 1b. *Loc. 1–Loc. 5* locations of trenches and fault outcrops

The M_w_ 6.2 (Mj 6.8) Nagano (Japan) earthquake of 22 November 2014 ruptured the Kamishiro Fault, which occurs along the northern segment of the Itoigawa–Shizuoka Tectonic Line (ISTL), the plate boundary between the Eurasian and North American plates in central Honshu Island, Japan (Fig. 1; Japan Meteorological Agency 2014; Lin et al. 2015a). Historical and instrumental records reveal that more than ten large earthquakes (M_w_ ≥ 6.0) have occurred in the vicinity of the northern segment of the ISTL during the last ca. 1200 years (Fig. 1b; Headquarters for Earthquake Research Promotion 2000). Historical documents also account for a large earthquake that occurred in AD 762 in the area around the Matsumoto Basin, but the magnitude is unknown and details on the hypocenter and extent of damage are unclear (National Astronomical Observatory of Japan 2015). Geologic and seismic data suggest that active faults developed on the eastern margins of the Matsumoto and Kamishiro basins have the potential to trigger large earthquakes of M_w_ >7.7–8.0 (Fig. 2; Headquarters for Earthquake Research Promotion 2000). Base on the trench investigations, a previous study has reported that the average recurrence interval of such earthquakes along the ISTL is ca. 3500–5000 years (e.g., Research Group for Ito-Shizu Tectonic Line Active Faults 1988). However, Okumura (2001) revealed four large-magnitude earthquakes occurring in the last 6500 years, with the most recent faulting event dated AD 841 on the northern segment of the ISTL, ~5 km south of the trench sites in this study (Fig. 3).

Our recent studies on the Kamishiro Fault that triggered the 2014 M_w_ 6.2 Nagano earthquake reveal (i) a 9.3-km long surface rupture zone with a thrust-dominated displacement characterized by distinct fault scarps with vertical offsets of up to 1.5 m, which are mostly duplicated on the pre-existing known Kamishiro Fault and an unknown active fault (Lin et al. 2015a), and (ii) at least three large earthquakes that are thought to be associated with surface ruptures of the Kamishiro Fault occurring in the last ca. 1200 years, exhibiting an average recurrence interval of ca. 300–500 years (Lin et al. 2015b).

To better understand the recent activity of the Kamishiro Fault, we conducted a paleoseismic study based on fault outcrop and trenche analysis. Here, we report on the results, including data on the recurrence interval and magnitudes of morphogenic earthquakes and Holocene slip rates of the Kamishiro Fault. We also discuss seismotectonic implications of the ISTL active fault system along the boundary between the Eurasian and North American plates.

## Tectonic setting

The study region is located along the eastern margins of the Matsumoto and Kamishiro basins in northern Nagano Prefecture, Honshu Island, Japan, along the northern segment of the ISTL (Fig. 1a). The 150 km-long ISTL active fault system, one of the most active fault systems in Japan, is considered to be the plate boundary between the Eurasian and North American plates (e.g., Kobayashi 1983; Nakamura 1983; Seno et al. 1993, 1996). South of the study area, the ISTL in the southern part of Honshu Island forms one arm of the triple junction between the Eurasian, North American, and Philippine Sea plates (Fig. 1a). Previous studies have shown that the ISTL is one of the most important active fault zones in central Japan, and that it represents a geological boundary that divides Honshu Island into western and eastern provinces (Fig. 1a) (Research Group for Active Faults of Japan, RGAFJ 1980, 1991).

The Kamishiro Fault is a major fault segment along the ISTL active fault system. It strikes generally N–S to NNE–SSW, dips at 30°–70° to the east, and is ~26 km long. The fault forms a topographical boundary, separating the Matsumoto and Kamishiro basins to the west from the Saigawa Hills to the east (Figs. 1b and 2; Research Group for Active Faults of Japan, RGAFJ 1980, 1991; Headquarters for Earthquake Research Promotion 2014). Basement rocks on the western side of the Matsumoto and Kamishiro basins are mainly pre-Neogene metamorphic rocks that include serpentinite mélange (Nakano et al. 2002). In contrast, basement rocks on the eastern sides of the basins are mainly Pliocene–Miocene sedimentary rocks overlain by unconsolidated alluvial and lacustrine deposits (Fig. 2).

Slip rates on different fault segments along the ISTL active fault system vary from 1.5 to 3.3 mm/a on the northern segment, the Kamishiro Fault (Research Group for Active Faults of Japan, RGAFJ 1980, 1991; Imaizumi et al. 1997; Okumura et al. 1998; Matsuta et al. 2001, 2004), which is the target segment in this study, to 5–14 mm/a on the central–southern segment (Ikeda and Yonekura 1986; Okumura 2001), and 5–8 mm/a on the southernmost segment (Lin et al. 2013a). Thrust slip rates of up to ~14 mm/a along the ISTL active fault system are the highest onland thrust fault slip rates reported in Japan (Research Group for Active Faults of Japan, RGAFJ 1980, 1991).

## Trench excavation and outcrop exposure

**Fig. 4:**
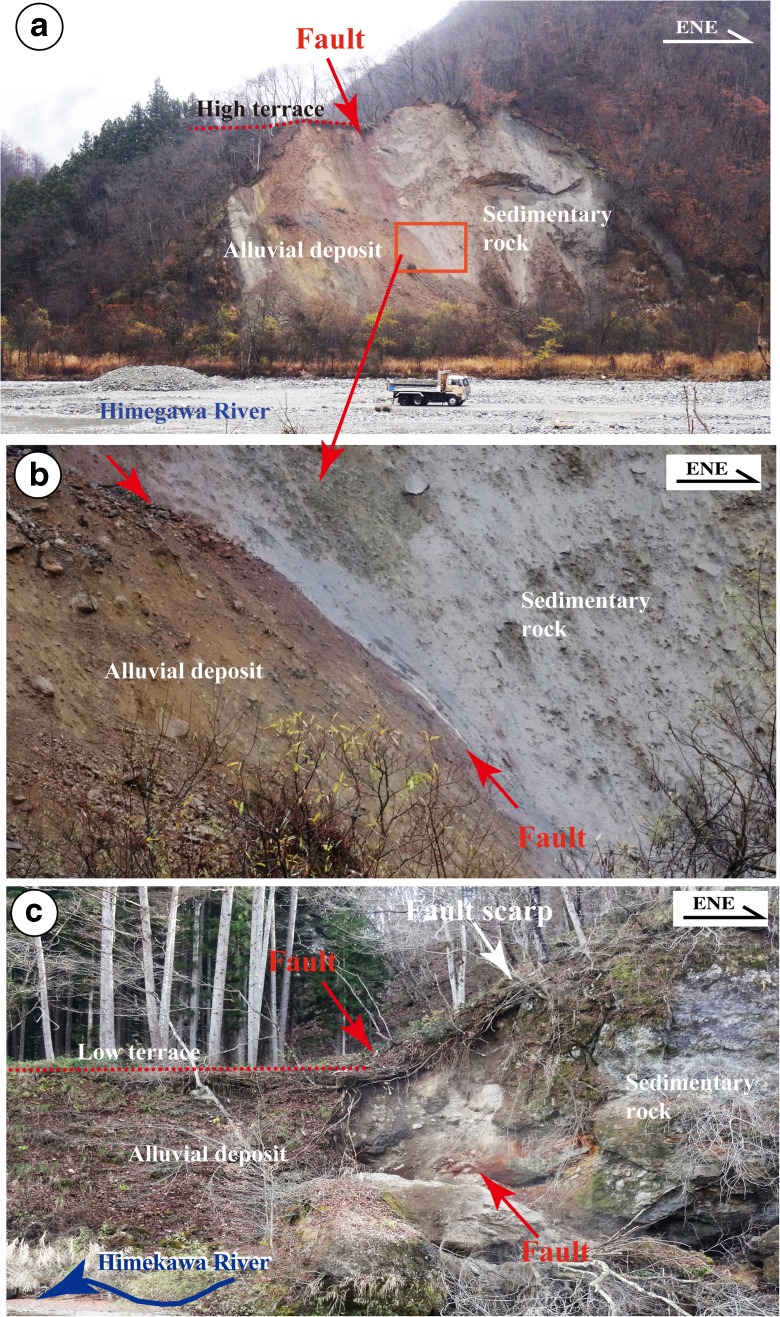
Representative fault outcrops showing thrust structures of the Kamishiro Fault at Locs. 1 and 2 (see Fig. 3 for locations). *Red arrows* indicate the fault plane. **a**, **b** Enlarged view of the fault outcrop at Loc. 1. Note that the Tertiary sedimentary rock is thrust over alluvial deposits of the high terrace riser. **c** Active fault scarp and outcrop at Loc. 2. Note that the Tertiary sedimentary rock is thrust over alluvial deposits of the low terrace riser

The 2014 Nagano earthquake produced a 9.3-km long co-seismic surface rupture zone coinciding with the previously mapped Kamishiro Fault trace along the ISTL. The surface-rupturing earthquake produced dominant thrusting with vertical offset amount of up to ~1.5 m (typical ~0.4–1.0 m) and subordinate right-lateral strike-slip displacement of ~0.6 m (Lin et al. 2015a, 2015b). Structures that developed during the co-seismic surface rupture zone include thrust faults, fault scarps, en-echelon cracks, folding structures such as mole tracks and flexural folds, and sand boils (Lin et al. 2015a, 2015b). Topographically, the surface trace of the Kamishiro Fault is curved, and it follows the geomorphological boundary between ranges and basins (Figs. 2 and 3). In the study area, the fault cuts both low and high terrace risers along the Himekawa River (Fig. 3). Along the east-dipping thrust faults, alluvial deposits of the terrace risers have been overthrusted by Neogene sedimentary rocks consisting of consolidated to weakly consolidated mudstone, sandstone, and volcanic tuff–breccia (Fig. 4). The geological and seismic reflection data show that the Kamishiro Fault cuts late Pleistocene-Holocene sedimentary strata (Research Group for Active Faults of Japan, RGAFJ 1980, 1991; Matsuta et al. 2001, 2004; Okumura et al. 1998).

**Fig. 5:**
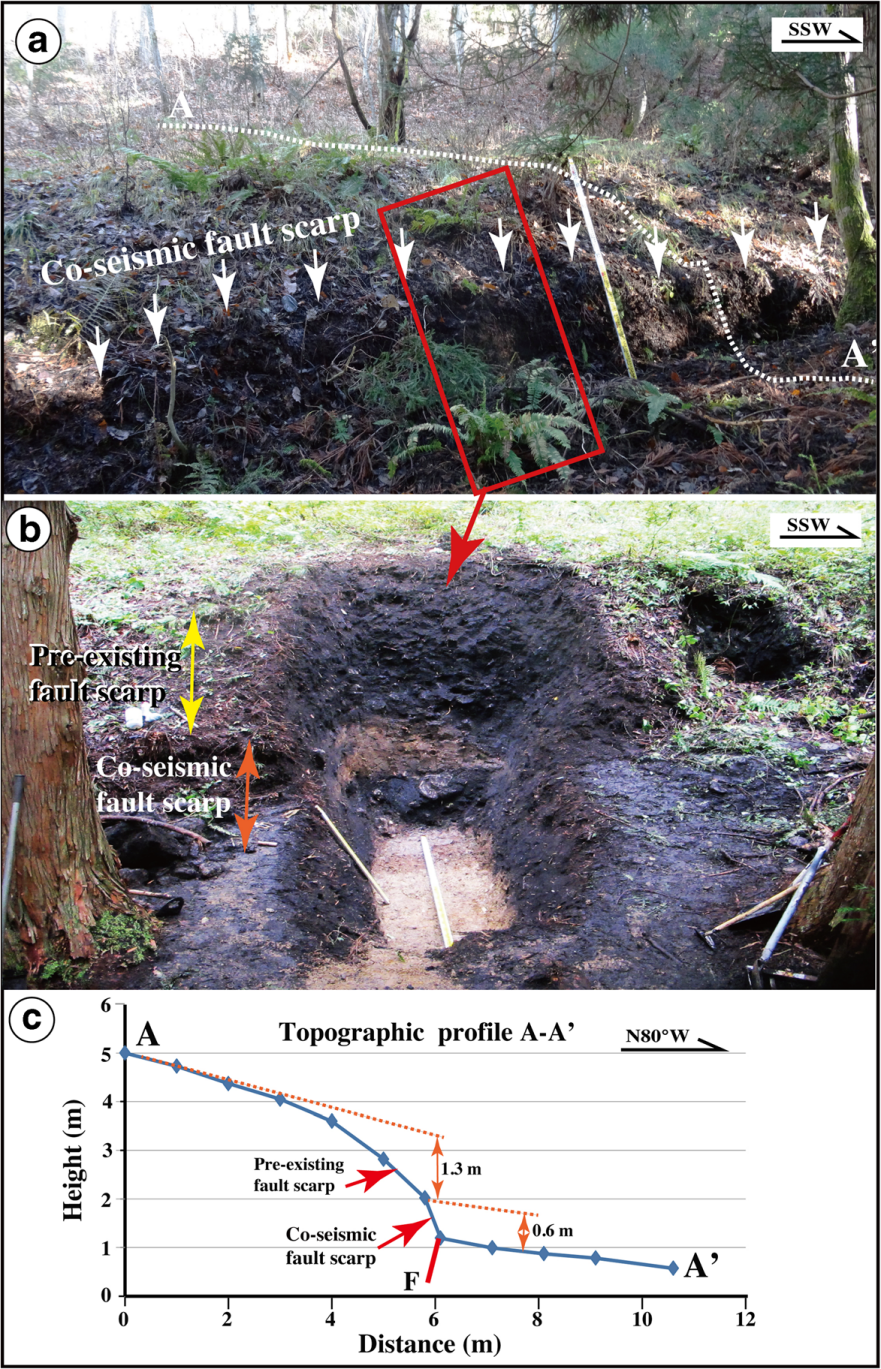
Photographs showing the Loc. 3 site and trench A. **a** Co-seismic fault scarp produced by the 2014 Nagano earthquake. **b** Overview of trench A dug across the fault scarp. **c** Topographic profile A–A’ across the 2014 co-seismic fault scarp. The fault scarp height produced by the 2014 earthquake is 0.6 m, and the cumulative throw of pre-existing fault scarp is 1.3 m

**Fig. 6:**
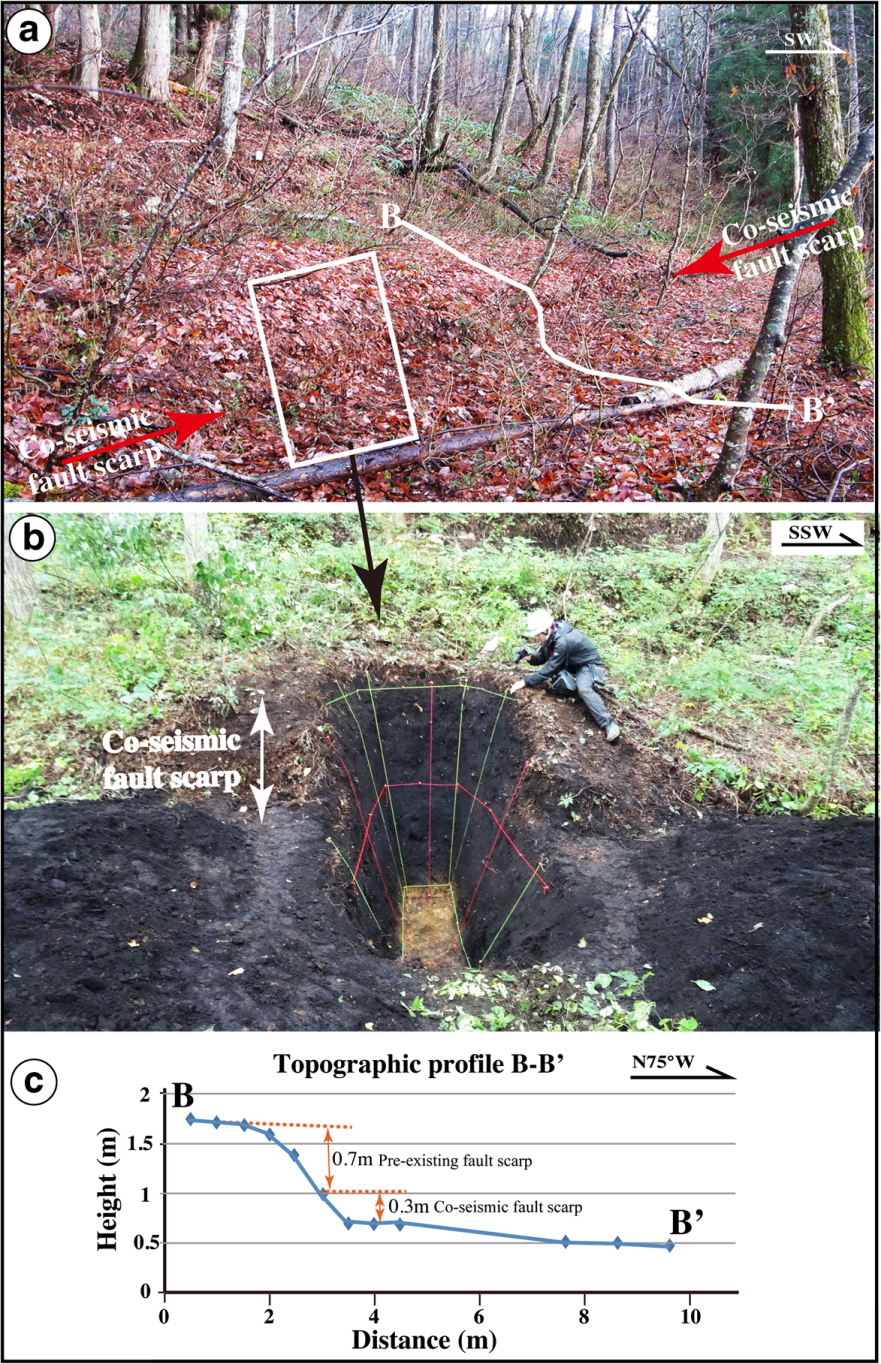
Photographs showing the Loc. 4 site and trench B. **a** Co-seismic fault scarp produced by the 2014 Nagano earthquake. **b** Overview of trench B across the fault scarp. **c** Topographic profile of B–B′ across the 2014 co-seismic fault scarp. The scarp height produced by the 2014 earthquake is 0.3 m, and the pre-existing fault scarp is 0.7 m height

**Fig. 7:**
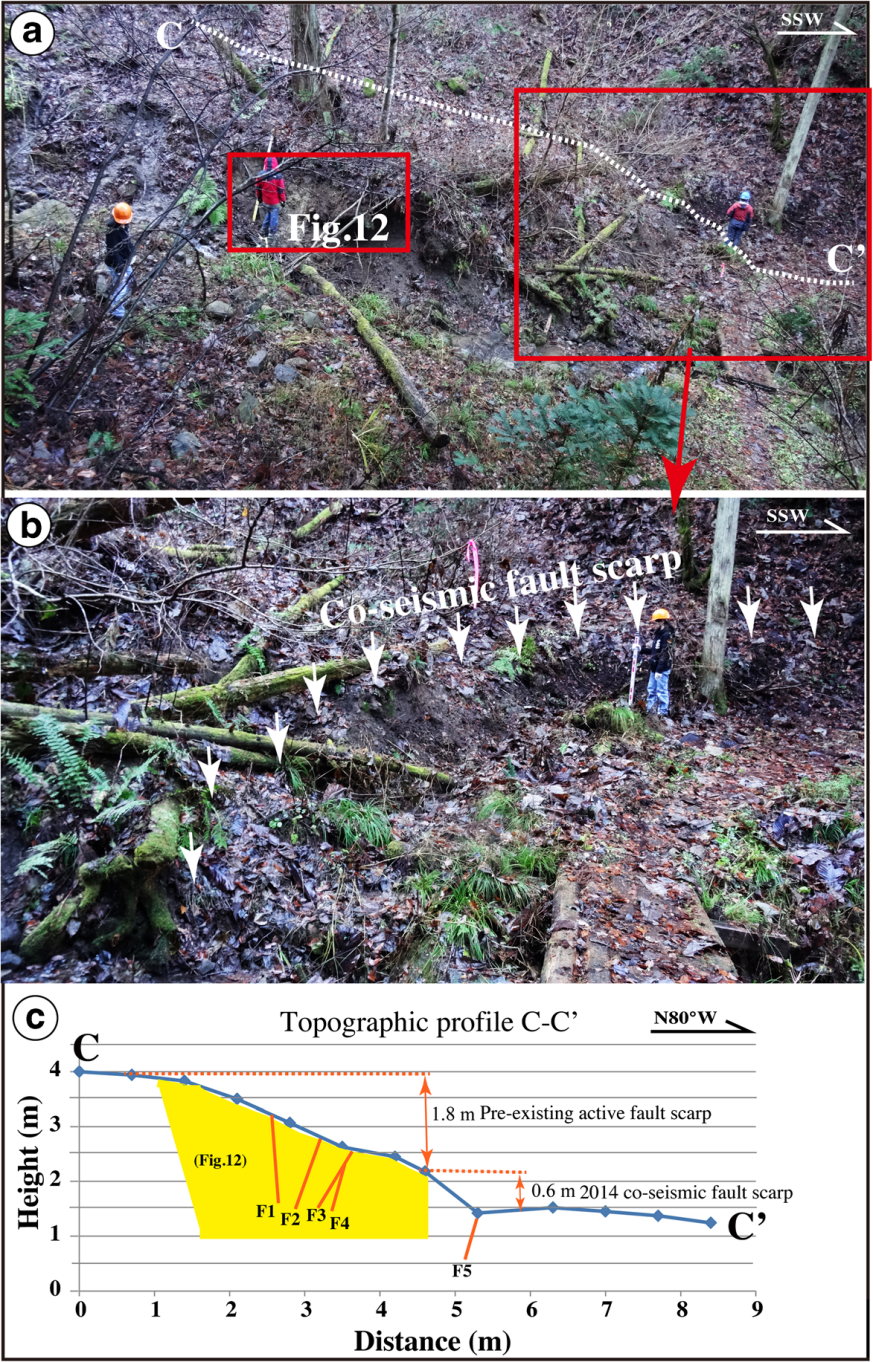
Photographs showing Loc. 5 site and the fault outcrop. **a** Co-seismic fault scarp produced by the 2014 Nagano earthquake. Location of the studied fault outcrop is indicated with the *red rectangle* (see Fig. 12 for detail). **b** Overview of the co-seismic fault scarp. **c** Topographic profile of C–C′. *Yellow area* and *red lines* are the faults across the 2014 co-seismic fault scarp. The scarp height produced by the 2014 earthquake is 0.6 m, and the cumulative throw is 1.8 m

In order to understand the recent activity of the Kamishiro Fault, including its paleoseismicity, we conducted trench excavations across the 2014 co-seismic fault scarp at two locations Locs. 3 and 4 in Fig. 3b (trenches A and B at Locs. 3 and 4, respectively; Fig. 3b) and excavated and cleaned one fault outcrop bounded by the 2014 co-seismic fault scarp at Loc. 5 (Fig. 3b) to observe fault structures in the unconsolidated fluvial and alluvial deposits and to collect samples for radiocarbon dating. All the three sites are located on lower terrace risers and alluvial fans developed along small valleys that are tributaries of the Himekawa River (Fig. 3), and the 2014 co-seismic fault scarps coincide with the locations of pre-existing fault scarps (Figs. 5, 6, and 7). All trenches and fault outcrop described in this study are located on a newly identified fault trace that coincides with the 2-km long co-seismic surface rupture zone produced by the 2014 M_w_ 6.2 earthquake (Fig. 3). Trenches and outcrop were excavated and smoothed by hand, as the study sites located in remote mountainous areas and were inaccessible to machinery. All topographical profiles were measured onsite by type measure.

The exposure walls of trenches and fault outcrop were sketched using a 1.0-m grid overlay and are described in detail below. Radiocarbon dating of the samples was performed via accelerator mass spectrometry (AMS) at the Institute of Accelerator Analysis, Japan (http://www.iaa-ams.co.jp/indexen.html). Dendrochronologically calibrated calendar ages were obtained using Stuiver et al. (2003). Radiocarbon dating results and calibrated ages are listed in Table 1.

**Table 1:** Results of ^14^C dating

Sample no.^a^	Lab no.	Material	^14^C age (years BP)^b^	Calendar year range (2σ)^c^
2014-C01	IAAA-141929	Organic soil	Modern	Modern
2014-C02	IAAA-141931	Organic soil	457 ± 23	AD 1418–AD 1456
2014-C03	IAAA-141932	Organic soil	193 ± 24	AD 1733–AD 1807
2014-C04	IAAA-141933	Organic soil	107 ± 24	AD 1806–AD 1930
2014-C05	IAAA-141934	Organic soil	1013 ± 24	AD 980–AD 1043
2014-C06	IAAA-141935	Organic soil	1592 ± 23	AD 413–AD 538
2014-C07	IAAA-141936	Organic soil	1748 ± 25	AD 234–AD 358
2014-C08	IAAA-141937	Organic soil	1599 ± 23	AD 405–AD 537
2015-C01	IAAA-151130	Organic soil	3568 ± 27	AD 1981–AD 1876
2015-C02	IAAA-151131	Organic soil	3733 ± 26	BC 2205–BC 2111
2015-C03	IAAA-151132	Organic soil	3419 ± 28	BC 1776–BC 1636
2015-C04	IAAA-151133	Organic soil	3156 ± 26	BC 1499–BC 1393
2015-C05	IAAA-151134	Organic soil	2168 ± 26	BC 359–BC 276
2015-C06	IAAA-151135	Organic soil	1492 ± 24	AD 538–AD 636
2015-C07	IAAA-151136	Organic soil	3096 ± 26	BC 1426–BC 1287
2015-C08	IAAA-151137	Organic soil	2318 ± 26	BC 411–BC 360
2015-C09	IAAA-151138	Organic soil	1633 ± 23	AD 377–AD 435
2015-C10	IAAA-151139	Organic soil	1260 ± 23	AD 671–AD 778
2015-C11	IAAA-151140	Organic soil	1643 ± 23	AD 340–AD 430
2015-C12	IAAA-151141	Organic soil	1672 ± 23	AD 331–AD 420
2015-C13	IAAA-151142	Organic soil	3669 ± 26	BC 2137–BC 1966
2015-C14	IAAA-151143	Organic soil	2981 ± 26	BC 1282–BC 1118
2015-C15	IAAA-151144	Organic soil	2159 ± 26	BC 648–BC 547
2015-C16	IAAA-151145	Organic soil	1440 ± 24	AD 577–AD 652
2015-C17	IAAA-151146	Organic soil	1635 ± 23	AD 377–AD 434
2015-C18	IAAA-151147	Organic soil	1702 ± 23	AD 317–AD 400
2015-C19	IAAA-151148	Organic soil	3603 ± 26	BC 2026–BC 1896
2015-C20	IAAA-151149	Organic soil	3554 ± 26	BC 1973–BC 1869
2015-C21	IAAA-151150	Organic soil	2059 ± 26	BC 166–AD 2
2015-C22	IAAA-151151	Organic soil	1761 ± 24	AD 215–AD 352
2015-C23	IAAA-151152	Organic soil	1150 ± 23	AD 801–AD 970
2015-C24	IAAA-151153	Organic soil	1121 ± 24	AD 879–AD 989
2015-C25	IAAA-151154	Organic soil	1080 ± 23	AD 941–AD 1017
2015-C26	IAAA-151155	Organic soil	1209 ± 24	AD 766–AD 888
2015-C27	IAAA-151156	Organic soil	1141 ± 24	AD 860–AD 977
2015-C28	IAAA-151157	Organic soil	4397 ± 29	BC 3774–BC 3653
2015-C29	IAAA-151158	Organic soil	5733 ± 29	BC 4624–BC 4499
2015-C30	IAAA-151159	Organic soil	4491 ± 29	BC 3347–BC 3092
2015-C31	IAAA-151160	Organic soil	3325 ± 27	BC 1683–BC 1529
2015-C32	IAAA-151161	Organic soil	2370 ± 24	BC 515–BC 392
2015-C33	IAAA-151162	Organic soil	2589 ± 26	BC 812 –BC 764
2015-C34	IAAA-151163	Organic soil	5659 ± 28	BC 4551–BC 4447
2015-C35	IAAA-151164	Organic soil	5470 ± 29	BC 4361– BC 4260
2015-C36	IAAA-151165	Organic soil	4577 ± 29	BC 3377–BC 3239
2015-C37	IAAA-151166	Organic soil	2333 ± 25	BC 430–BC 364
2015-C38	IAAA-151167	Organic soil	2243 ± 25	BC 318–BC 207
2015-C39	IAAA-151168	Organic soil	2514 ± 26	BC 694–BC 542
2015-C40	IAAA-151169	Organic soil	5986 ± 29	BC 4948–BC 4792
2015-C41	IAAA-151170	Organic soil	5889 ± 28	BC 4831–BC 4707
2015-C42	IAAA-151171	Organic soil	5712 ± 29	BC 4619–BC 4462
2015-C43	IAAA-151172	Organic soil	5611 ± 30	BC 4500–BC 4361

^a^All samples were analyzed at the Institute of Accelerator Analysis Ltd., Japan, via accelerator mass spectrometry (AMS)

^b^Radiocarbon ages were measured using accelerator mass spectrometry referenced to the year AD 1950. Analytical uncertainties are reported at 2σ

^c^Dendrochronologically calibrated calendar age by method A from CALIB Radiocarbon Calibration version 6.1 (Stuiver et al. 2003)

### Trenches

#### Trench A

**Fig. 8:**
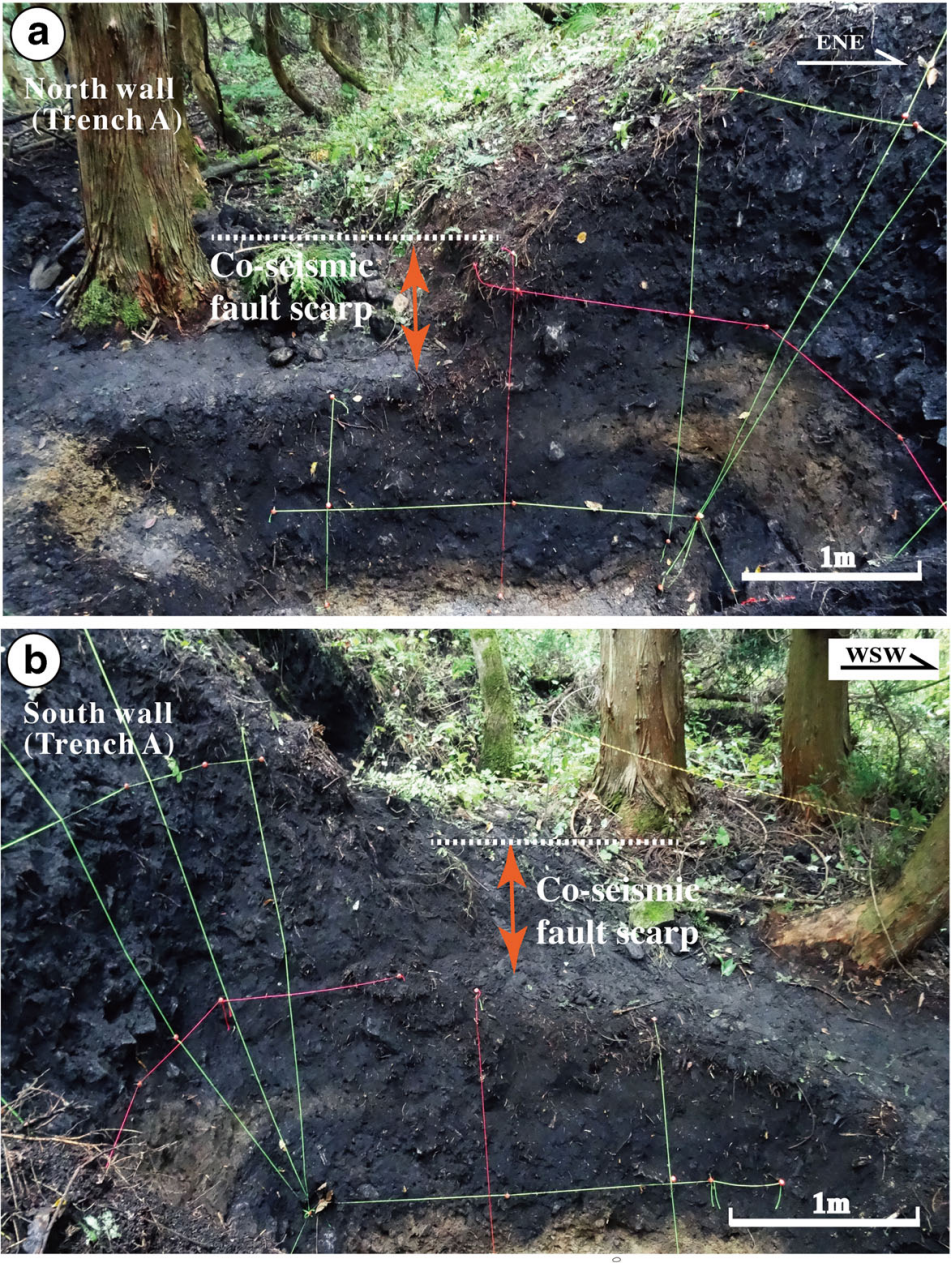
Photographs showing the north (**a**) and south (**b**) walls of Trench A at Loc. 3

**Fig. 9:**
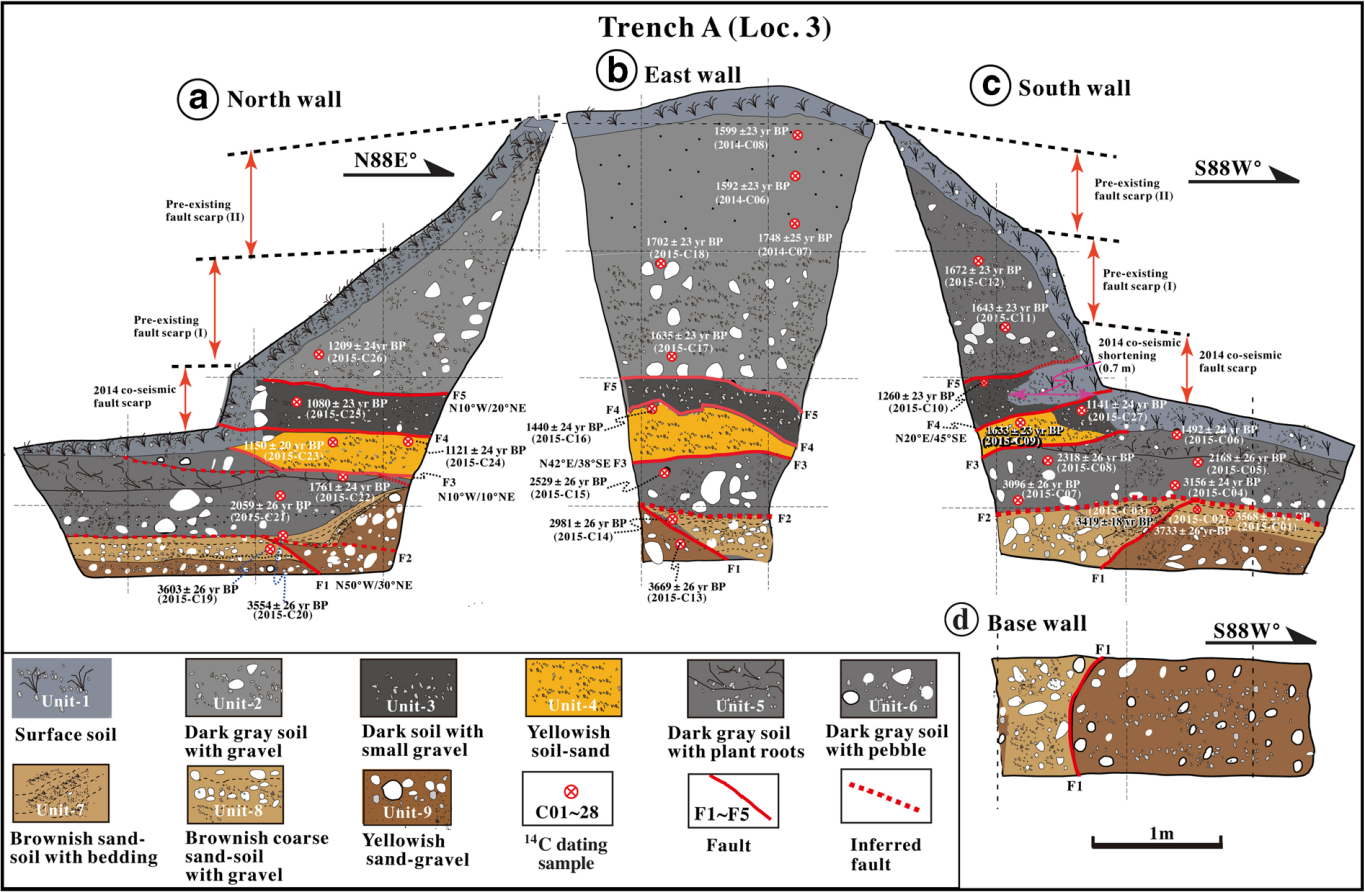
Sketches of the north (**a**), east (**b**), and south (**c**) walls in trench A. Note that the old soil materials of unit 2, which yield ^14^C ages of 1210–1748 years BP, overlie the younger soil and soil–sand layers of units 3 and 4, which yield ^14^C ages of 1080–1150 years BP

Trench A was excavated on a lower terrace riser across the 2014 co-seismic fault scarp (Loc. 3, Fig. 5). The unconsolidated deposits exposed in this trench are mainly surficial debris and alluvial deposits that can be divided into nine sedimentary units (units 1–9) based on their nature, properties, and relation (Figs. 8 and 9). Units 1–3, 5, and 6 are mainly composed of gray to dark gray surficial soil with some gravel and pebbles sourced from alluvial and/or fluvial deposits. Unit 4 is composed of yellowish gray alluvial soil–sand, bedded (Fig. 8). Units 7–9 are composed of gray to yellowish gray stratified coarse sand–gravel and sand–soil alluvial deposits with bedding structures.

Five faults (F1–F5) were identified in trench A, putting in tectonic contact units 2–9 (Fig. 9). The 2014 co-seismic slip occurred along fault F4, where sediments of unit 3 are juxtaposed against overlying modern surficial soil deposits (Fig. 9a, c). At this trench location, the vertical offset and horizontal shortening along fault F4 caused by the 2014 earthquake are 0.6 and 0.7 m, respectively.

The ^14^C dating results show that unit 2 formed at 1209–1748 years BP, which overlie units 3 and 4 along fault F5, is older than units 3 and 4 formed at 1080–1260 and 1150–1633 years BP, respectively, indicating that at least one reverse faulting event occurred along faults F4 and F5 (Fig. 9 and Table 1; see Sect. 5 for details).

The fault scarp shows a sharp slope change, from a high angle at the base of the scarp to a low angle at the upper part (Fig. 9). The fault scarp has been continuously eroded after its formation, and the sharp change in slope angle on the scarp indicates a composite scarp associated with repeated surface faulting events. Such morphologic change of fault scarp has also been reported along many active faults in the world (e.g., Wallace 1977; Zhang et al. 1986). The scarp morphology indicates that at least two faulting events prior to the 2014 earthquake occurred after the formation of the alluvial deposits in the study area (see Sect. 5 for details).

#### Trench B

**Fig. 10:**
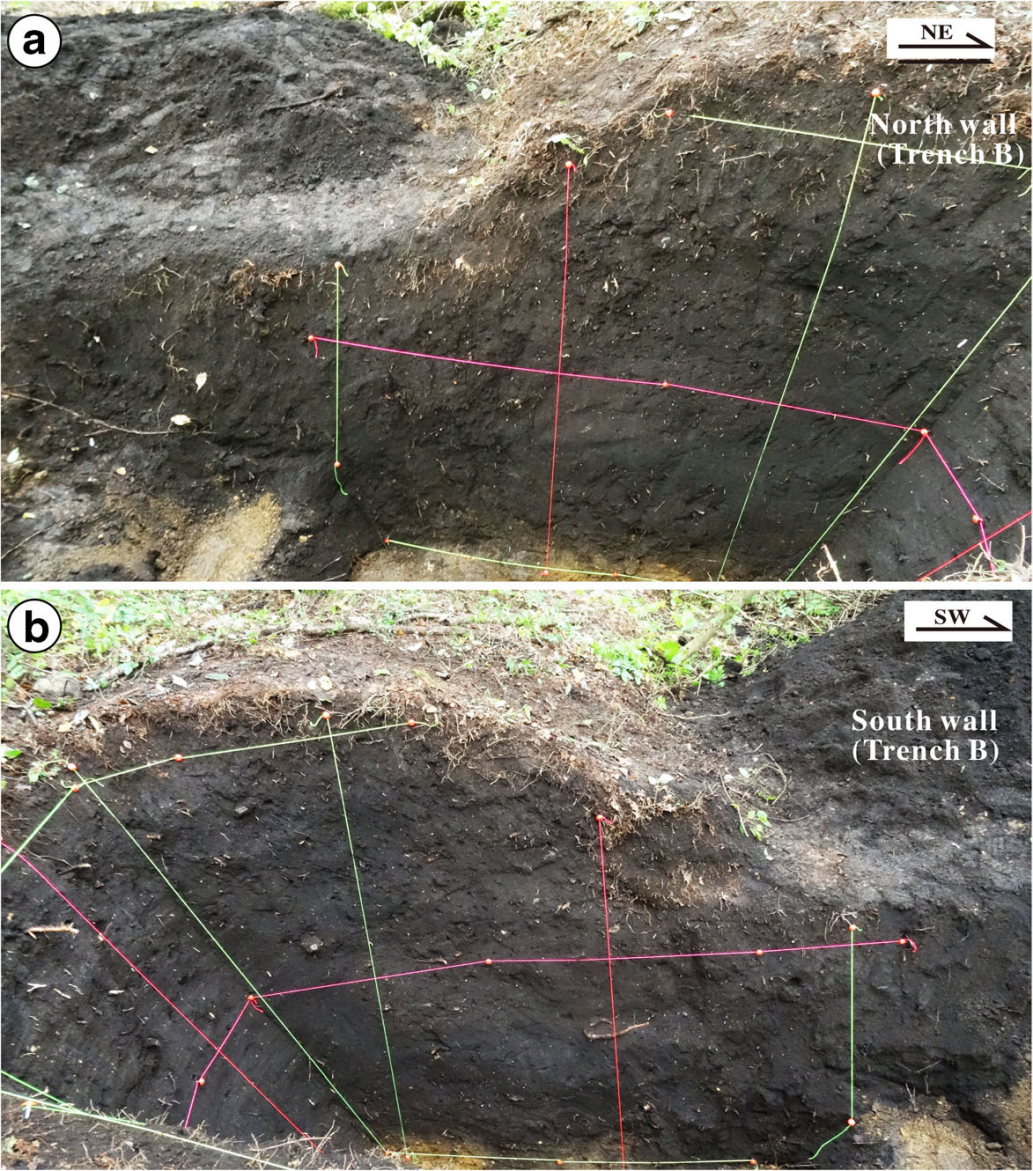
Photographs showing the north (**a**) and south (**b**) walls of trench B at Loc. 4

**Fig. 11:**
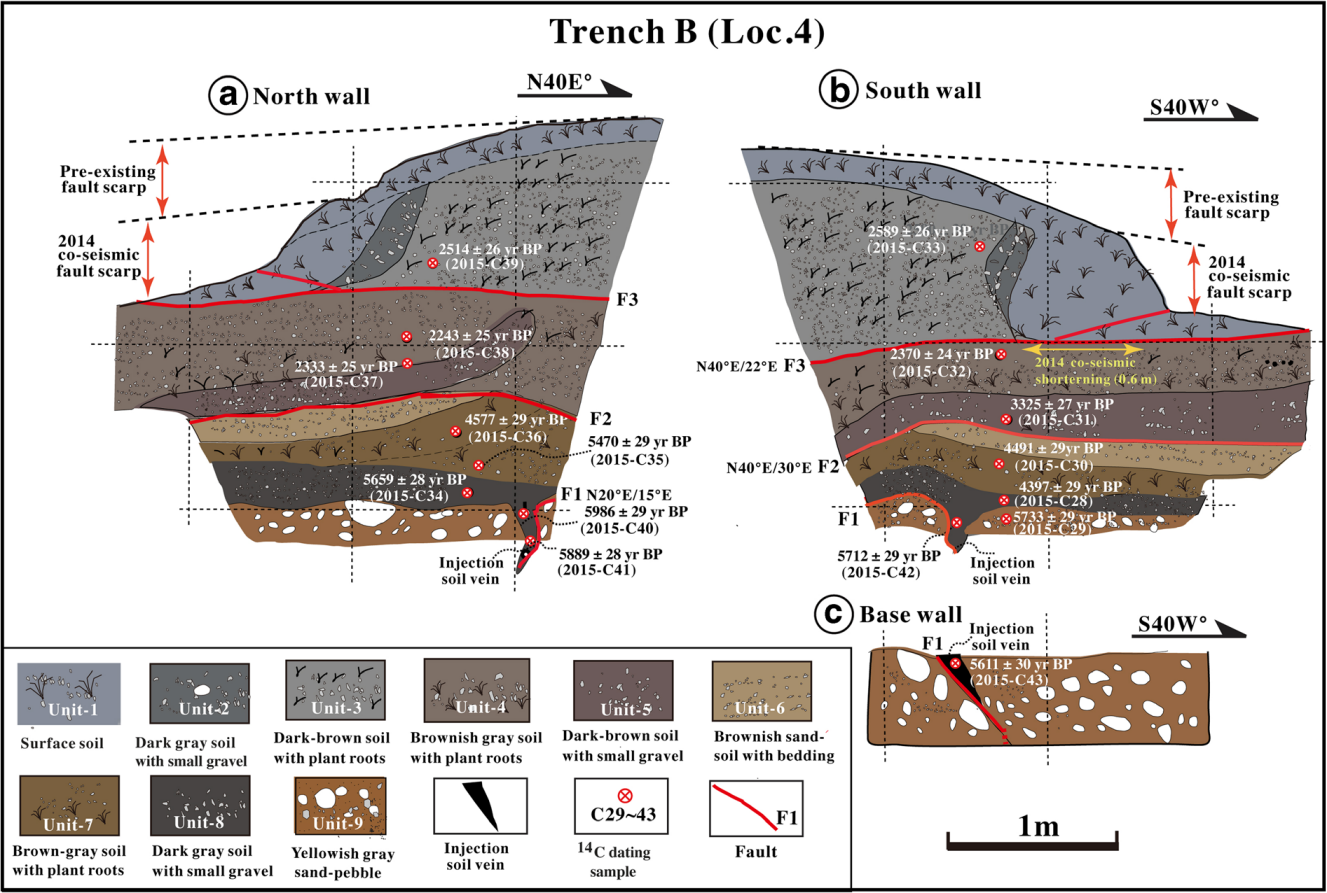
Sketches of the north (**a**) and south (**b**) walls in trench B at Loc. 4. Note that the soil materials of unit 3, with ages of 2514–2589 years BP, overlie the younger soil and soil–sand layers of units 4 and 5, yielding ages of 2243–2370 years BP

Trench B is located ~550 m south of trench A at Loc. 4 (Fig. 10). Similar to trench A, the unconsolidated deposits exposed in this trench are mainly composed of surficial debris and alluvial material and can be divided into nine sedimentary units based on their nature, properties, and fault relation (Figs. 10 and 11). Units 1–4 consist of surficial soil that contains numerous plant roots and some small gravels. Units 1–3 are gray to dark gray in color, and unit 4 is brownish gray to dark brown. Unit 5 consists of brownish gray soil with numerous small gravel layers. Unit 6 is composed of stratified sand–soil with bedding structure that overlies the older brown–gray surficial soil (unit 7) with numerous weakly carbonized plant roots. Unit 8 consists of dark gray soil with some small gravel layers that overlie and are partially injected into the sand–pebble layers of unit 9 as a wedge-shaped vein (Fig. 11).

Three main faults (F1–F3) are identified in trench B. At this location, the 2014 co-seismic slip event occurred along fault F3, showing a vertical offset of 0.3–0.6 m (Figs. 6 and 11; Lin et al. 2015a). Radiocarbon ages of organic soil show that the sediments of unit 3 formed at 2514–2589 years BP, prior to the formation of the underlying unit 4 (2243–2370 years BP), units 5–7 (2333–5470 years BP), and the alluvial deposits of units 8 and 9 (5660–5996 years BP) (Fig. 11 and Table 1). The juxtaposition of the older unit 3, on units 4 and 5 indicates that a thrusting event occurred during the formation of unit 4 at 2243–2370 years BP. The soil veins of unit 8 injected into unit 9 along fault F1 suggest that a faulting event occurred during the period between the formation of unit 7 (4491–5470 years BP) and unit 8 (4397–5659 years BP) (see Sect. 5 for details).

### Fault outcrop

**Fig. 12:**
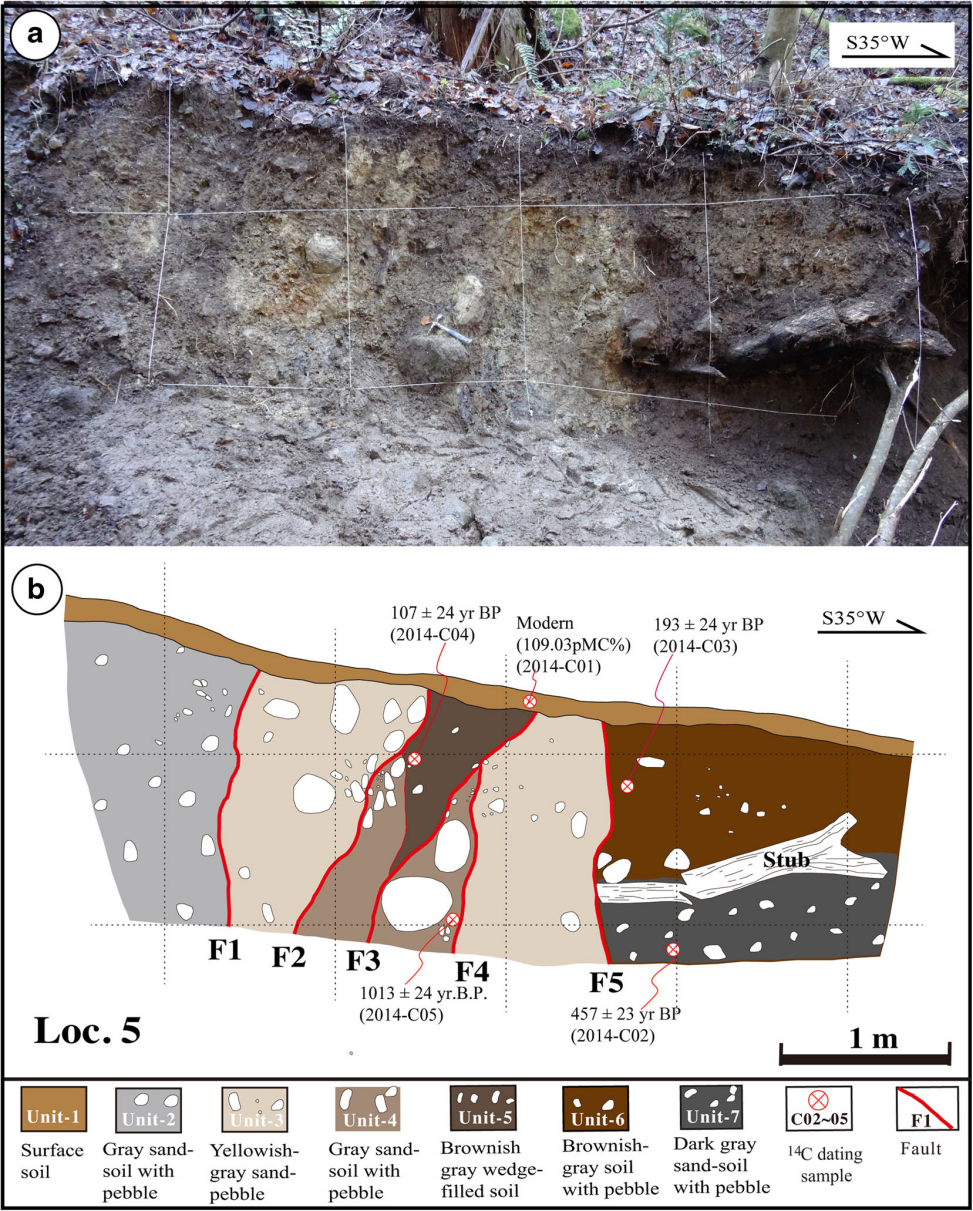
Photograph (**a**) and accompanying sketch (**b**) of the fault outcrop at Loc. 5 (see Fig. 3), showing the faults

Numerous fault outcrops were identified along the Kamishiro Fault, and one of the most representative fault outcrops was better exposed and cleaned (Fig. 12). The outcrop is exposed at Loc. 5, located between Loc. 3 and Loc. 4 (in Fig. 3) in a small river channel, just 2 m east of the 2014 co-seismic fault scarp (Fig. 7). The sediments, which are fluvial deposits lacking any bedding, can be divided into seven units. Unit 1 is composed of present-day gray to dark gray surficial soils, while units 2–7 consist of unconsolidated sand–pebble and gray to dark gray sand–soil deposits.

Five faults identified in the outcrop wall form the sharp and vertical contact between the sediment units (Fig. 12). Radiocarbon ages show that units 5 and 6 formed at 107 and 193 years BP, respectively (Fig. 12 and Table 1), and that the deposits of units 4 and 7 formed at 1013 and 457 years BP, respectively (Fig. 12 and Table 1).

## Identification of linear morphogenic faulting events

**Fig. 13:**
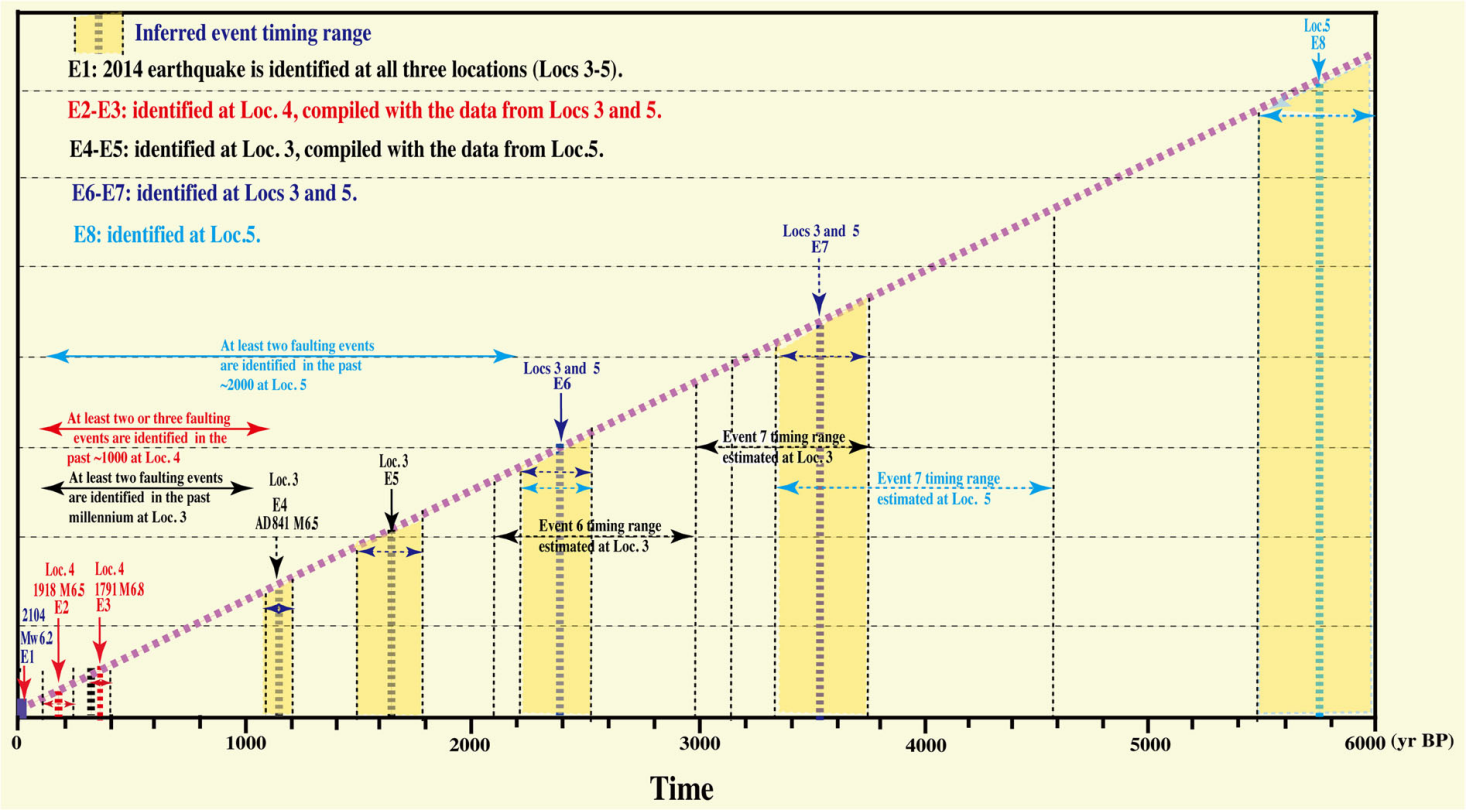
Timing of seismic events inferred upon the Kamishiro Fault, including the 2014 earthquake (E1) and seven late Holocene historic/paleoearthquakes (E2–E8) identified from offset features and deformed alluvial sediments and terrace risers. Three events likely correspond to historically recorded earthquakes identified in the last ca. 1200 years, suggesting a recurrence interval of earthquakes along the Kamishiro Fault of 300–400 years during the past 200 years

Linear morphogenic earthquakes are large-magnitude earthquakes that are capable of generating or modifying the surface morphology instantaneously and permanently (Caputo 2005). It is well known that large earthquakes of magnitude >6–7 with shallow focal depths can produce distinctive co-seismic surface ruptures and cause strong ground deformation (Yeats et al. 1997; Lin et al. 2010, 2015a, 2015b). Co-seismic surface ruptures and ground deformation structures are generally characterized by distinctive landforms, for example, characterized by horizontal and vertical offsets of topographic markers such as streams, gullies, mountain ridges, flexure-folds of the ground surface, fault scarps, and surficial sediments. Geomorphological and field investigations of such features are relatively straightforward and can yield considerable information regarding fault mechanisms and the mechanics of large earthquakes. Co-seismic surface ruptures produced by the 2014 Nagano earthquake (E1) were observed both at the two trenches and at the fault outcrop analyzed in this study. Based on the observed topographic features and geological structures including faults and deformation of sediments, we identified seven linear morphogenic earthquakes (E2–E8) that occurred prior to the 2014 Nagano earthquake (E1); three of these correspond to historically recorded earthquakes that occurred in the study area, as discussed below (Fig. 13).

### Event 2

At Loc. 5, all the sedimentary units exposed in the fault outcrop formed during the last millennium. Unit 5 is the youngest deposit (excepting the modern soil) that is composed of wedge-shaped fill and in tectonic contact with unit 4. Units 4 and 5 are bounded by faults F2–F4 (Fig. 12). This setting suggests that the most recent faulting event prior the 2014 earthquake occurred during the formation of unit 5, during the last 107 years BP (AD 1806–AD 1930; Table 1). Historical records show that an M_w_ 6.5 earthquake occurred on 11 November 1918 at the southern end of the Kamishiro Fault (Fig. 1b; Headquarters for Earthquake Research Promotion 2000), causing extensive damage and producing a 1-km long surface rupture zone in the northern Nagano Prefecture (Utsu et al. 1987; National Astronomical Observatory of Japan 2015). The geodetic and seismic analyses show that the seismogenic fault of the 1918 earthquake is a thrust fault striking N15°E and dipping east at angle of 75° (Tada and Hashimoto 1988), coincident with the Kamishiro Fault. We, thus, infer that this historical event corresponds to the most recent faulting event (E2) recognized in the fault outcrop.

### Event 3

A penultimate faulting event (E3) identified at Loc. 5 within the fault outcrop wall occurred during the period between 193 and 457 years BP (AD 1733–1807 and AD 1418–1456; Table 1). The loose soil of unit 6 overlies the weakly consolidated sand–soil of unit 7, with an age gap of ca. 250 years (Fig. 12). Both units are fault-bounded and are in contact with unit 3 by fault F5. We infer that the sediments of unit 6 were sourced from a kind of colluvial material (formed at 193 years BP; Table 1) that overlies unit 7, on account of collapse of the F5 fault scarp after the formation of unit 7, possibly suggesting the occurrence of a morphogenic earthquake that occurred right before the formation of unit 6 (193 years BP or AD 1733–1897). Historical records describe a severe damaging following an M_w_ 6.8 earthquake in the southern area of the Kamishiro Fault on 23 July 1791 (Fig. 1b; Headquarters for Earthquake Research Promotion 2000; Utsu et al. 1987; Tokyo Astronomical Observatory, 2015). Accordingly, we suggest that the penultimate seismic faulting event identified in this study could be associated with the 1791 M_w_ 6.8 earthquake.

Based on the structural features observed in the exposed wall and topographic profile measured at Loc. 5, the amount of vertical offset across faults F1–F5 is 1.8 m, while the co-seismic vertical offset related to the 2014 event was 0.6 m (Figs. 7 and 12). These data indicate that the pre-existing fault scarp of 1.8 m is likely cumulative of two or more faulting events with magnitudes comparable to the 2014 earthquake. These older events occurred after formation of the alluvial deposits of unit 4 (1013 years BP; Table 1). An accumulation of vertical offset caused by multiple faulting events is also observed at Loc. 3, where the vertical offset on a pre-existing fault scarp is 1.3 m, while the 2014 co-seismic vertical offset is 0.6 m (Fig. 5). Furthermore, the slope angle in the upper part of the pre-existing fault scarp becomes gentler above a knick point (Fig. 9). Radiocarbon ages reveal that the fault scarp formed after the formation of sediments during the period of 1260–1018 years BP (AD 671–1017, Table 1).

The horizontal component of slip observed at trench A along faults F4 and F5 is estimated to be >1.5 m, while during the 2014 M_w_ 6.2 earthquake, it was only 0.7 m (fault F4). These data show that at least one slip event along fault F4, prior to the 2014 earthquake, occurred after the formation of unit 4 (1013–1121 years BP or AD 801–AD 989; Table 1).

Based on the observed topographic features and geological structures, as well as the ^14^C ages, we infer that two or three morphogenic events occurred prior to the 2014 earthquake in the last ca. 1000–2000 years at Locs. 3 and 5; these events probably correspond to the E2 and E3 events.

### Event 4

The third event back (E4) inferred at Loc. 3 occurred during the period of 1080–1260 years BP, during the formation of unit 3 (AD 941–1017 and AD 671–778; Table 1). Unit 2, which yields ^14^C ages of 1502–1748 years BP, overlies the younger unit 3, which yield ages of 1080–1260 ± 20 years BP. Seven organic soil samples of unit 2 taken from the hanging wall of fault F5 yield consistent radiocarbon ages in the range of 1502–1748 years BP (Table 1), except for sample 2015-C26 (1210 years BP), which was collected at a shallower depth near the present-day surface; these dates are older than those of units 3 and 4 in the footwall of fault F5. These ages show that unit 2 overthrust the younger sediments of units 3 and 4 after the formation of unit 3, indicating that a seismic faulting event occurred during the period of 1080–1260 years BP (AD 671–AD 1017). Alternatively, the soil–sand layers of unit 4 unconformably overlie the older surficial soil materials of unit 5 and alluvial deposits of unit 6 along fault F3 (Fig. 9), suggesting that a faulting event occurred sometime during the period of formation of unit 4 (1150–1635 years BP or AD 377–AD 970) and unit 5 (1402–1761 years BP or AD 352–AD 636, Table 1).

Historical documents record a strong earthquake that caused extensive damage in the northern Nagano Prefecture on 3 August AD 841 (Utsu et al. 1987). Based on the damage distribution, this earthquake is inferred to have occurred on the active fault system of the ISTL, and its magnitude is estimated to have been 6.5 < M_w_ < 7.0 (Headquarters for Earthquake Research Promotion 1996). Accordingly, we suggest that one event (E4) corresponds to the AD 841 earthquake.

### Event 5

At Loc. 3, the alluvial sediments of unit 4 unconformably overlie an old surficial soil (unit 5). This yellowish soil–sand layer of unit 4 is irregularly fault-bounded and overlain and underlain by dark-gray soil and soil with gravel layers (units 5 and 3, respectively). Based on the sediments and fault structures, we infer that a faulting event (E5) occurred during the period of 1492–1761 years BP (AD 538–AD 352), during the formation of the old dark-gray surficial soil of unit 5 (Fig. 9 and Table 1).

The topographic profile at Loc. 3 (Fig. 5) shows that a 0.6-m high co-seismic fault scarp caused by the 2014 earthquake coincides with the location of a 1.3-m high pre-existing fault scarp. The slope angle of the pre-existing fault scarp changes from 40° in the lower part of the scarp to 20° in the upper part. Considering the change in slope of the fault scarp and the amount of vertical offset caused by the 2014 earthquake, we infer that two faulting events occurred at this site after the formation of the old surficial soil of unit 5 (1492–1761 years BP or AD 215–AD 636; Table 1), with magnitudes similar to that of the 2014 earthquake.

### Event 6

At Loc. 4, the radiocarbon ages of the soil deposits of unit 3 (2514–2589 years BP or BC 542–BC 812 ) are older than those of the brownish-gray soil layers of unit 4 (2243–2370 years BP or BC 318–BC 515; Table 1). Unit 4 is mainly composed of brownish-gray soil with minor amounts of weakly carbonized plant roots and is comparable in color and composition to the overlying soil deposits of unit 3. The 2014 M_w_ 6.2 earthquake caused horizontal shortening of 0.4–0.6 m across fault F3 (Lin et al. 2015a; Fig. 11), with a horizontal displacement of >2 m (Fig. 11). The occurrence of old soil overlaying younger soil and the structural features show that the soil deposits of unit 3 were thrust upon those of unit 4, indicating that at least one thrusting event occurred during the period of 2243–2370 years BP (BC 207–BC 515) after the formation of unit 4.

The topographic profiles at Loc. 4 also show the presence of a pre-existing step-shaped fault scarp with a change in slope angle at a knick-point (Figs. 6 and 11). The older vertical offset of 0.6–0.7 m is similar to that caused by the 2014 Nagano earthquake. This topographic feature also reveals that a fault-scarp-forming event occurred after the formation of unit 3. Therefore, we infer that at least one faulting event (E6) occurred after the formation of unit 4 (2243–2370 years BP or BC 207–BC 515).

At Loc. 3, the deposits of unit 6, which formed during 2059–3156 years BP (BC 166–BC 1499), unconformably overlie the alluvial deposits of units 7 and 8 (Fig. 9; Table 1). Based on the structural and sedimentary features of the deposits of unit 6, we tentatively infer that the boundary between units 6 and 7 is a fault (F2), along which the deposits of unit 6 were thrust over unit 7. This relationship suggests that a faulting event occurred during the formation of unit 6, during the period of 2059–3156 years BP (BC 166–BC 1499), possibly corresponding to event E5 recognized at Loc. 5.

### Event 7

At Loc. 4, the deposits of unit 5, which formed at 3325 years BP, unconformably overlie (across fault F2) the sand–soil layers of unit 6 and the older surficial soil of unit 7 (4491–5470 years BP or BC 4260–BC 3092) (Fig. 11 and Table 1). On the northeast sides of both the northern and southern trench walls, the deposits of units 6 and 7 are cut and deformed by fault F2, across which the thickness of deposits changes sharply. Based on these structural features, we infer that a faulting event (E7) occurred after the formation of unit 5, in the period BC 1683–BC 1529 (3325 years BP).

The E7 faulting event is also identified at Loc. 3, where alluvial deposits of unit 7, which formed during the period of 2981–3733 years BP (BC 1118–BC 2205), are cut and folded by fault F2 and are overlain by alluvial deposits of the lower part of unit 6, which yields radiocarbon ages of 3096–3156 years BP (BC 1499–BC 1287, sample nos. 2015-C04 and 2015-C07). These relationships indicate that a faulting event occurred during the period of 2981–3733 years BP (BC 1118–BC 2205). Considering the error range of radiocarbon ages, we suggest that this event is associated with faulting event E7, as inferred at Loc. 4 (see above).

### Event 8

Event E8 is identified at Loc. 4, where the dark-gray soil of unit 8, which yields ages of 5611–5986 years BP (BC 4361–BC 4948, sample nos. 2015-C34, and 2015-C40 to 2015-C43; see Fig. 11), is injected into the underlying sand–pebble alluvial deposits of unit 9. Unit 8 is covered by old surficial soils of unit 7 which formed at 4591–5470 years BP (BC 4260–BC 3092). This injection vein of soil materials indicates that a faulting event occurred in the period of 5611–5986 years BP (BC 4361–BC 4948), after the formation of unit 8 and prior to the formation of unit 7 (Fig. 11). After the formation of unit 9, the sand–pebble alluvial fan deposits were uplifted, and the old surficial soils of unit 7, which are brown–gray in color and contain numerous weakly carbonized plant roots, formed. Event E8 is the oldest faulting event identified in this study.

## Discussion

### Recurrence interval of morphogenic earthquakes

Historic and paleoseismic studies show that recurrence intervals of morphogenic earthquakes can be relatively well constrained, thus providing the most direct measure of past recurrence intervals of moderate to large earthquakes along present-day active faults (e.g., Yeats et al. 1997; MaCalpin 2009; Lin et al. 2015a). Historical and instrumental records show that three large earthquakes (M_w_ ≥ 6.0) have occurred in the region close to the Kamishiro Fault, in the vicinity of the northern Matsumoto Basin, during the last 300 years (Fig. 1b; M_w_ 6.3 in 1714 and M_w_ 6.5 and 6.1 in 1918) (Headquarters for Earthquake Research Promotion 2000). The 1918 M_w_ 6.5 Omachi earthquake deformed the ground surface along a steeply dipping active fault in the Matsumoto Basin (Tada and Hashimoto 1988). Trench investigation by Okumura (2001) reported that the recurrence interval of large earthquakes on the northern section of the ISTL around the study area has been ca. 1500 years during the last ca. 6000 years, and that the most recent event probably occurred in AD 841.

As described above, we identified seven seismic faulting events that occurred prior to the 2014 earthquake (E1) in the last ca. 6000 years. Three of these events (E2–E4), occurred in the last ca. 1200 years and have been associated with historically recorded earthquakes: the 1918 M_w_ 6.5, 1791 M_w_ 6.8, and AD 841 M_w_ 6.5 earthquakes. No historical records are associated with the older events (E5–E7); they probably occurred during the period of ca. 1200–3800 years (Fig. 13). The recurrence intervals of the identified events (E1–E4) corresponding to historically recorded earthquakes, including the 2014 earthquake, are variable from ca. 100 to ca. 700 years, with an average range l of 300–400 years, while the recurrence intervals of events E5–E8 range from ca. 800 to ca. 2800 years. The longer recurrence intervals of the older events may be related to a lack of geological evidence, as well as possible hiatuses in the record of historical events in the study area, which is rather limited. Our results are in contrast with a previous estimate of the recurrence interval of surface-rupturing earthquakes of ca. 1500 years during the last ca. 6000 years (Okumura 2001; Headquarters for Earthquake Research Promotion, 2010). The recurrence interval inferred for the Kamishiro Fault in the present study is much shorter than the recurrence intervals for the main intracontinental active faults in Japan, which range from ca. 1000 to more than 10,000 years and are typically ca. 2000–4000 years (RGAFJ, 1991). However, the figure for the Kamishiro Fault is comparable to the 150–500-year recurrence interval along the Fujikawa-kako Fault Zone in the southern segment of the ISTL active fault system (Lin et al. 2013a) and is also comparable to the ca. 50–400-year recurrence interval for subduction-zone earthquakes along the Suruga and Sagami troughs (Fig. 1a; Headquarters for Earthquake Research Promotion, 2010). These observations indicate that the occurrence of morphogenic earthquakes along both the northern and southern segments of the ISTL is characterized by a relatively short time span of 300–500 years, showing a recurrence interval comparable with subduction-zone-type earthquakes, suggesting that the Kamishiro Fault is an active fault developed on the plate boundary between the Eurasian and North American plates, which is the onland extension of the subduction zone along the Suruga Trough (Fig. 1a; Lin et al. 2013a).

### Possible magnitudes of morphogenic earthquakes and seismotectonic implications

Geological and seismic reflection data suggest that the active faults that bound the eastern margins of the Matsumoto and Kamishiro basins have the seismic potential to trigger earthquakes of M_w_ >7.7–8.2 (Headquarters for Earthquake Research Promotion 2000). However, historical records show that the magnitudes of four recent earthquakes, including the 2014 Nagano earthquake, that have occurred on the Kamishiro Fault during the last ca. 1200 years are <7 (i.e., 2014 M_w_ 6.2, 1918 M_w_ 6.5, 1791 M_w_ 6.8, and AD 841 M_w_ 6.5).

Topographic features show that the vertical offsets caused by individual historically recorded earthquakes identified in this study are 0.5–1.0 m (Figs. 9 and 11), similar to the offset produced by the 2014 M_w_ 6.2 earthquake (0.4–1.0 m) (Lin et al. 2015a), indicating similar magnitudes for these identified earthquakes that have occurred during the last ca. 1200 years. This finding is consistent with the offsets mentioned in historical records. Two questions emerge from these observations: (1) why are the magnitudes of the identified historical earthquakes limited to M_w_ <7 and (2) which factors play a role in determining the characteristic magnitudes of earthquakes occurring on the Kamishiro Fault?

Geological data show that basement rocks in the study area and bounded by the Kamishiro Fault are mainly Paleozoic serpentinized mélange (Nakano et al. 2002). Serpentine is closely associated with transform faults (e.g., Christensen 1972; Francis 1981; Moore and Rymer 2007) and with seismogenic segment of subduction zones (Ulmer and Trommsdorff 1995); indeed, the presence of serpentine minerals is considered to facilitate creep along the San Andreas Fault (Moore and Rymer 2007), thereby explaining its low fault strength (Wibberley 2007). The thermal pressurization of fluid released by the dehydration of serpentine and friction melt also plays an important role in the dynamic weakening of faults during seismic slip within seismogenic fault zones (Wibberley and Shimamoto 2005; Lin et al. 2013b). High-speed experimental results confirm that the serpentine dehydration that accompanies frictional melting results in a sudden increase in pore pressure, which in turn may lead to a reduction in the effective normal stress across the fault, in turn resulting in a marked reduction in the dynamic fault strength, thereby enabling further slip along the fault in a subduction zone (Lin et al. 2013b). Previous studies have shown that the magnitudes of many historical and paleoearthquakes on the central and southern segments of the ISTL, where no serpentinate is present, are on the order of M_w_ ~8.0 (Headquarters for Earthquake Research Promotion 2000; Lin et al. 2013a), which is in contrast to the smaller magnitudes of linear morphogenic earthquakes occurring along the Kamishiro Fault. The presence of serpentinate within the Kamishiro Fault may be a principal factor that weakens the fault strength and prevents the accumulation of strain energy on the seismogenic fault zone sufficient to trigger a large-magnitude earthquake. We therefore consider that the characteristic magnitude of earthquakes along the Kamishiro Fault (M_w_ <7) is constrained mainly by the weakness of the fault zone due to the presence of serpentinate along this segment of the ISTL. Accordingly, we may expect that the potential for the Kamishiro Fault to trigger a large earthquake of M_w_ >7.7–8.2 in the next 100 years is very low, as inferred by the Headquarters for Earthquake Research Promotion (2000).

### Slip rate of the Kamishiro Fault

In order to estimate long-term slip rates, we used organic soil materials in old surficial soil layers and alluvial deposits for radiocarbon dating, yielding 52 uniform ages (Table 1 and Figs. 9, 11, and 12). Based on these dating results and the vertical offsets on fault scarps, we estimated the throw rate for the Kamishiro Fault. As shown in Figs. 7 and 9, the vertical offset of fault scarps observed at Locs. 3 and 5 is 2.0–2.5 m, and surficial soil materials and alluvial deposits at the base of the fault scarps yielded ^14^C ages of ca. 1000 years BP (1080–1150 and 1013 years BP, respectively). The average throw rate is therefore calculated to be 2.0–2.5 mm/a during the last millennium. Based on the recurrence interval of 300–500 years and the characteristic offset amount of 0.5–1.0 m produced by individual morphogenic earthquakes, as documented above, the throw rate is estimated to be 1.0–3.3 mm/a (average, ~2.1 mm/a), which is comparable to the long-term slip rate of 1.5–3.3 mm/a during the Pleistocene–Holocene, as estimated from topographic and geological data in the study area along the Kamishiro Fault (Research Group for Active Faults of Japan, RGAFJ 1980, 1991; Imaizumi et al. 1997; Okumura et al. 1998; Matsuta et al. 2001). The slip rate of 2–2.5 mm/a for the Kamishiro Fault, in the northern segment of the ISTL, is in contrast to slip rates of 5–14 mm/a in the central–southern segment (Ikeda and Yonekura 1986; Okumura 2001) and 5–8 mm/a in the southernmost segment (Lin et al. 2013a). As compared with the slip rates on the central–southern segment of the ISTL, the lower slip rate and smaller magnitude of characteristic earthquakes on the Kamishiro Fault could be possibly accounted for by the presence of serpentinate within the seismogenic fault zone.

As compared with active intraplate faults on Honshu Island, Japan, the relatively shorter recurrence intervals for moderate-stronger earthquakes on the Kamishiro Fault indicate that present-day activity on this fault is closely related to seismic faulting along the plate boundary between the Eurasian and North American plates, as has occurred on the central–southern segments of the ISTL, which represent the onland extension of the subduction zone along the Suruga Trough.

## Conclusions

The following conclusions can be drawn on the basis of field investigations, trench excavations, and radiocarbon dating results:

1. We identified at least seven large Holocene paleoearthquakes on the Kamishiro Fault prior to the 2014 M_w_ 6.2 Nagano earthquake. Three of these events are likely associated with historically recorded earthquakes of the last ca. 1200 years, having their macro-seismic epicenters in the area of the Kamishiro Fault. The resulting average recurrence interval is ca. 300–400 years on the seismogenic Kamichiro Fault in the past 1200 years.
2. The relatively lower earthquake magnitudes on the Kamishiro Fault (M_w_ < 7) may be caused by weakening of the seismogenic fault zone due to the presence of serpentinate.
3. The most recent linear morphogenic earthquake (E2) prior to the 2014 earthquake (E1) corresponds to the 1918 M_w_ 6.5 earthquake. This penultimate faulting event (E3) occurred during the period of AD 1800–1400 and was probably associated with the 1791 M_w_ 6.8 earthquake. The antepenultimate faulting event (E4) is inferred to have occurred during the period of AD 500–1000, probably corresponding to the AD 841 M_w_ 6.5 earthquake.
4. The slip rate on the Kamishiro Fault ranges between 1.0 and 3.3 mm/a, with an average of ~2.1 mm/a.

Our findings differ from those of previous studies (Okumura 2001; Headquarters for Earthquake Research Promotion, 2010) that reported a recurrence interval for morphogenic earthquakes of ca. 1500 years. Our results reveal that the style and magnitude of thrust displacements are constrained mainly by a weak fault zone, caused by the presence of serpentinate within the fault and along the ISTL which represents the plate boundary between the Eurasian and North American plates.

## References

[CR1] Caputo R (2005). Ground effects of large morphogenic earthquakes1: preface. J Geodyn.

[CR2] Christensen NJ (1972). The abundance of serpentinites in the oceanic crust. J Geophys Res.

[CR3] Francis TJG (1981). Serpentinization faults and their role in the tectonics of slow spreading ridges. J Geophys Res.

[CR4] Geospatial Information Authority of Japan (2014a) Information of epicentral area of the northern Nagano earthquake. http://www.gsi.go.jp/BOUSAI/h26-nagano-earthquake-index.html. Accessed 20 Feb 2016

[CR5] Headquarters for Earthquake Research Promotion (1996) Investigation results and evaluation of the Itoigawa-Shizuoka Tectonic Line active fault system. http://jishin.go.jp/main/chousa/96augit/index.htm. Accessed 20 Feb 2016

[CR6] Headquarters for Earthquake Research Promotion (2000) Evaluation of fault zone geometry of the Itoigawa-Shizuoka Tectonic Line. www.jishin.go.jp/main/kyoshindo/01a/tenpu1.pdf.Accessed 20 Feb 2016

[CR7] Headquaters for Earthquake Research Promtion (2010) Evaluation of fault zone geometry of the Itoigawa-Shizuoka Tectonic Line. http://www.jishin.go.jp/main/kyoshindo/o1a/tenpu1.pdf. Accessed 20 Feb 2016

[CR8] Headquarters for Earthquake Research Promotion (2014) Characteristics of the 2014 Nagano earthquake. http://www.jishin.go.jp/main/yosokuchizu/chubu/p20_nagano.html. Accessed 20 Feb 2016

[CR9] Ikeda Y, Yonekura N (1986). Determination of late quaternary rates of slip on two major fault zones in central Japan. Bulletin of the Earthquake Research Institute, University of Tokyo.

[CR10] Imaizumi, T., Haraguchi, T., Nakata (other 8) (1997). Slip rate on the Kamishiro active fault along the northern part of the Itoigawa-Shizuoka Tectonic Line, detected by long-geo-slicer and drilling. Active Fault Research 16:5–43 (in Japanese with English abstract).

[CR11] Japan Meteorological Agency (2014) Earthquake information. http://www.jma.go.jp/en/quake/20141122221109395-222208.html. Accessed 20 Feb 2015

[CR12] Kobayashi Y (1983). Initiation of subduction Japan. Geogr Rev Jpn.

[CR13] Lin A, Ren Z, Dong J (2010) Co-seismic ground-shortening structures produced by the 2008 M_w_ 7.9 Wenchuan earthquake, China. Tectonophysics 491:21–34

[CR14] Lin A, Iida K, Tanaka H (2013). On-land active thrust faults of the Nankai-Suruga subduction zone: the Fujikawa–kako Fault Zone, central Japan. Tectonophysics.

[CR15] Lin A, Takano S, Hirono T, Kanagawa K (2013). Coseismic dehydration of serpentinite during large earthquakes: evidence from high-velocity friction experiments. Chem Geol.

[CR16] Lin A, Mikako S, Yan B, Wang M (2015). Co-seismic surface ruptures produced by the 2014 M_w_ 6.2 Nagano earthquake, along the Itoigawa-Shizuoka Tectonic Line, central Japan. Tectonophysics.

[CR17] Lin A, Mikako S, Yan B, Wang M (2015b) Preliminary study of paleoseismicity on the Kamishiro fault that triggered the 2014 M_w_ 6.2 Nagano earthquake. Abstract, No.: 01341, 2015 Annual meeting of Japan earth and planetary science union

[CR18] MaCalpin JP (2009) Paleoseismology. 2nd edn. Academic Press, p 613

[CR19] Matsuta N, Ikeda Y, Imaizumi T, Sato H (2001). Subsurface structure of and rate of the net slip on the Kamishiro fault, northern part of the Itoigawa-Shizuoka tectonic line, central Japan. Active Fault Research.

[CR20] Matsuta N, Ikeda Y, Sato H (2004). The slip rate along the northern Itoigawa-Shizuoka tectonic line active fault system, central Japan. Earth Planet Space.

[CR21] Moore DE, Rymer MJ (2007). Talc-bearing serpentinite and the creeping section of the San Andreas fault. Nature.

[CR22] Nakamura K (1983). Possibility of a nascent plate boundary at the eastern margin of the Japan Sea. Bulletin of Earthquake Research Institute, University of Tokyo.

[CR23] Nakano S, Takeuchi M, Yoshikawa T, Nagamori H, Kariya Y, Okumura K, Taguchi Y (2002). Geology of the Shiroumadake district. Quadrangle Series, 1:50,000, Geological Survey of Japan, pp105 (in Japanese with English abstract)

[CR24] National Astronomical Observatory of Japan (2015). Rikanenpyou.

[CR25] Okumura K (2001). Paleoseismology of the Itoigawa-Shizuoka tectonic line in Central Japan. J Seismol.

[CR26] Okumura K, Imura R, Imaizumi T, Togo M, Sawa H, Mizuno K, Kariya Y (1998). Recent surface faulting events along the northern part of the Itoigawa-Shizuoka tectonic line-trenching surveys of the Kamishiro Fault and East Matsumoto Basin faults, ventral Japan. Zisin, the Journal of Seismological Society of Japan.

[CR27] Research Group for Active Faults of Japan (RGAFJ) (1980). Active faults in Japan—sheet maps and inventories.

[CR28] Research Group for Active Faults of Japan (RGAFJ) (1991). Active faults in Japan—sheet maps and inventories.

[CR29] Research Group for Ito-Shizu Tectonic Line Active Faults (1988). Late quaternary activities in the central part of Itoshizu tectonic line excavation study at Wakamiya and Osawa faults, Nagano prefecture, central Japan. Bulletin of the Earthquake Research Institute, University of Tokyo.

[CR30] Seno T, Stein S, Griff AE (1993). A model for the motion of the Philippine Sea plate consistent with NUVEL-1 and geological data. J Geophys Res.

[CR31] Seno T, Sakurai T, Stein S (1996). Can the Okhotsk plate be discriminated from the north American plate?. J Geophys Res.

[CR32] Stuiver M, Reimer PJ Reimer R (2003) CALIB Radiocarbon Calibration Version 4.4. http://radiocarbon.pa.qub.ac.uk/calib/. Accessed 20 Feb 2015

[CR33] Tada T, Hashimoto M (1988). A fault model of the 1918 Omachi, central Japan earthquake and its tectonic significance. Zisin, the Journal of Seismological Society of Japan.

[CR34] Tokyo Astronomical Observatory (2015) Chronological Scientific Tables. Muruzen Publishing Co. Ltd, Tokyo, Japan

[CR35] Ulmer P, Trommsdorff V (1995). Serpentine stability to mantle depths and subduction–related magmatism. Science.

[CR36] Utsu T, Yoshi T, Yamashina K, Shima E (1987). Encyclopedia of earthquake.

[CR37] Wallace RE (1977). Profiles and ages of young fault scarps, Northcentral Nevada. Geol Soc Am Bull.

[CR38] Wibberley CAJ (2007). Seismology: talc at fault. Nature.

[CR39] Wibberley CAJ, Shimamoto T (2005). Earthquake slip weakening and asperities explained by thermal pressurization. Nature.

[CR40] Yeats RS, Sieh K, Allen CR (1997). The geology of earthquake. Oxford University Press, pp 557

[CR41] Zhang B, Liao Y, Guo S, Wallace RE, Bucknam RC, Hanks TC (1986). Fault scarps related to the 1739 earthquake and seismicity of the Yinchuan graben, Ningxia Huizu Zizhiqu. China Bull Seism Soc Am.

